# Chemokine CCL21 determines immunotherapy response in hepatocellular carcinoma by affecting neutrophil polarization

**DOI:** 10.1007/s00262-024-03650-4

**Published:** 2024-02-17

**Authors:** Wenxin Xu, Jialei Weng, Minghao Xu, Qiang Zhou, Shaoqing Liu, Zhiqiu Hu, Ning Ren, Chenhao Zhou, Yinghao Shen

**Affiliations:** 1 Department of Liver Surgery and Transplantation, Liver Cancer Institute, Zhongshan Hospital, Fudan University, Key Laboratory of Carcinogenesis and Cancer Invasion, Ministry of Education, Shanghai, 200032 People’s Republic of China; 2 Key Laboratory of Whole-Period Monitoring and Precise Intervention of Digestive Cancer of Shanghai Municipal Health Commission, Shanghai, 201199 People’s Republic of China; 3 Department of Hepatobiliary and Pancreatic Surgery, Minhang Hospital, Fudan University, Shanghai, 201199 People’s Republic of China; 4 Institute of Fudan-Minhang Academic Health System, Minhang Hospital, Fudan University, Shanghai, 201199 People’s Republic of China

**Keywords:** CCL21, Hepatocellular carcinoma, Tumor microenvironment, Immunotherapy, Neutrophil polarization, PD-1

## Abstract

**Background:**

The efficacy of immune checkpoint inhibitors (ICIs) in hepatocellular carcinoma (HCC) is poor and great heterogeneity among individuals. Chemokines are highly correlated with tumor immune response. Here, we aimed to identify an effective chemokine for predicting the efficacy of immunotherapy in HCC.

**Methods:**

Chemokine C‐C motif ligand 21 (CCL21) was screened by transcriptomic analysis in tumor tissues from HCC patients with different responses to ICIs. The least absolute shrinkage and selection operator (LASSO) regression analysis was conducted to construct a predictive nomogram. Neutrophils in vitro and HCC subcutaneous tumor model in vivo were applied to explore the role of CCL21 on the tumor microenvironment (TME) of HCC.

**Results:**

Transcriptome analysis showed that CCL21 level was much higher in HCC patients with response to immunotherapy. The predictive nomogram was constructed and validated as a classifier. CCL21 could inhibit N2 neutrophil polarization by suppressing the activation of nuclear factor kappa B (NF-κB) pathway. In addition, CCL21 enhanced the therapeutic efficacy of ICIs.

**Conclusion:**

CCL21 may serve as a predictive biomarker for immunotherapy response in HCC patients. High levels of CCL21 in TME inhibit immunosuppressive polarization of neutrophils. CCL21 in combination with ICIs may offer a novel therapeutic strategy for HCC.

**Supplementary Information:**

The online version contains supplementary material available at 10.1007/s00262-024-03650-4.

## Introduction

Liver cancer is one of the leading causes of tumor-related deaths worldwide, with the sixth highest incidence and third highest mortality rate currently [1]. Over 90% cases of primary liver cancers are hepatocellular carcinomas (HCC), and life expectancy following a diagnosis of HCC is lower than for many other cancers [2]. One factor contributing to the poor prognosis is that the majority of patients have advanced HCC or metastases by the time they are diagnosed, missing the opportunity for therapeutic resection [3]. In recent years, the advent of targeted therapies and immunotherapies like immune checkpoint inhibitors (ICIs) has changed the treatment regimen for advanced or unresectable HCC, but objective remission rates remain poor [4]. Tumor heterogeneity is a key factor leading to immunotherapy failure as well as drug resistance [5]. Evaluating tumor heterogeneity can help to improve the efficacy of HCC immunotherapy. Therefore, it is imperative to seek effective biomarkers for accurately predicting the efficacy of HCC immunotherapy and novel interventional therapies.

Chemokines are defined as a superfamily of cytokines with chemotactic and pro-inflammatory properties that induce directed chemotaxis and regulate immune cell migration during inflammatory responses [6]. Numerous studies have shown that chemokine levels are highly correlated with tumor progression, tumor immune response, and prognosis. For example, in breast cancer, the expression of C-X-C motif chemokine ligand 9 (CXCL9) correlates with an increase in the number of tumor-infiltrating lymphocytes, and high levels of CXCL9 suggest a prolonged survival of patients [7–9]. Therefore, chemokines are potentially valuable biomarkers for predicting immunotherapy response in cancer.

Immune cell infiltration within the tumor microenvironment (TME) is critical for the efficacy of immunotherapy [10]. Tumor-associated neutrophils (TANS), as an important component of the TME, are classified into N1 and N2 types, and play a key role in tumor immunotherapy. It was found that N1 neutrophils exhibited anti-tumor effect and N2 neutrophils exhibited immunosuppressive properties [11]. Therefore, exploring the polarization and regulation mechanisms of neutrophils in the TME will contribute to improving the efficacy of tumor immunotherapy.

Here, we analyzed the transcriptome data of tumor tissues from 10 HCC patients with different response to immunotherapy and found that there was significant upregulation of CCL21 expression in the HCC tissues of patients that responded to immunotherapy. Besides, HCC patients with high serum CCL21 levels were more sensitive to immunotherapy. Subsequently, we constructed a CCL21-based nomogram for HCC immunotherapy response prediction. In addition, we found that CCL21 affects the TME landscape of HCC characterized by inhibition of N2 neutrophil polarization and promotion of CD8^+^ T-cell infiltration, which makes HCC more sensitive to anti-programmed death-1 (PD-1) therapy in the preclinical model. This study provides important theoretical support and clinical guidance for the application of CCL21 in individualized immunotherapeutic strategies in HCC.

## Materials and methods

### Parents and specimens

For ELISA, we collected 96 pre-treatment serum samples from HCC patients treated with anti-PD-1 antibodies at Zhongshan Hospital, Fudan University, Shanghai, China, between May 2020 and January 2021. The diagnosis of HCC was established through conventional imaging methods, with or without elevated serum tumor markers, following guidelines of the American Association for the Study of Liver Diseases (AASLD) or the Chinese National Liver Cancer (CNLC) [12, 13]. The inclusion criteria of patients included: (1) patients who presented with advanced-stage HCC, did not meet the up-to-seven criteria, or had inadequate remnant liver volume (less than 40% of standard liver volume for those with cirrhosis, or less than 30% for non-cirrhotic individuals) [14]; (2) patients who did not receive locoregional treatment before surgery, such as transarterial chemoembolization (TACE) or hepatic artery infusion chemotherapy; (3) patients who did not receive other types of monotherapy or combination regimens. The responders group defined as patients who assessed as complete response (CR) or partial response (PR) for more than 6 months according to the modified Response Evaluation Criteria in Solid Tumors (mRECIST) version 1.1. The clinicopathological variables were obtained from the electronic medical record system. The study protocol was approved by the research ethics committee of Zhongshan Hospital, Fudan University and performed in accordance with the principles set by the Declaration of Helsinki. All patients signed written informed consent for therapy and study inclusion.

### Construction and assessment of the signature-based nomogram

The least absolute shrinkage and selection operator (LASSO) regression analysis was conducted to construct the predictive signatures with 1000-fold cross-validation using the ‘glmnet’ package in R software. Independent predictive factors determined by LASSO regression analysis of cohort were integrated to construct the nomogram using the “rms” package in R software. We next performed the calibration curve and receiver operating characteristic (ROC) analysis to assess the predictive accuracy of nomogram. Additionally, the value of the nomogram for clinical applications was assessed by quantifying the net benefit using decision curve analysis (DCA).

### Analysis of public databases

The RNA sequencing data of the cancer genome atlas liver hepatocellular carcinoma (TCGA-LIHC) cohort was downloaded from the Genomic Data Commons Data Portal. Transcriptome data was normalized using the ‘limma’ package in R software before further analyses. The tumor immune infiltration and activity enrichment scores for each sample were assessed by performing single-sample gene set enrichment analysis (ssGSEA) in R software using the ‘gsva’ package. In addition, the absolute infiltration proportions of various immune cell types were calculated using cell-type identification by estimating relative subsets of RNA transcripts (CIBERSORT) algorithm.

### Cell isolation and culture

Peripheral blood was collected from healthy volunteers into EDTA-coated tubes. First, neutrophils were isolated using MojoSort™ Whole Blood Human Neutrophil Isolation Kit (Cat. 480,152, Biolegend). Aliquot 1 mL of human whole blood into a 5 mL (12 × 75 mm) polypropylene tube and add 10 µL of the Biotin-Antibody Cocktail. After incubating on ice for 15 min, add 10 µL of Streptavidin Nanobeads and incubate on ice for 15 min. Subsequently, wash the cells by adding MojoSort™ Buffer up to 4 mL and centrifuge the cells at 300×g for 5 min. Remove supernatant by Pipet aid instead of pouring and then add 3 ml MojoSort™ Buffer. Pour out the unlabeled fraction and pool the unlabeled fractions, which were neutrophils. The obtained neutrophils were resuspended in RPMI 1640 medium supplemented with 10% fetal bovine serum, 1% penicillin, and streptomycin, seeded in 6-well plates. Subsequently, to investigate the influence of CCL21 on neutrophil functional polarization, we stimulated neutrophils with 100 ng/ml recombinant human CCL21 (Cat. 300-35A, PeproTech) for 3 h and then used for subsequent experiments.

### RNA isolation and transcriptome sequencing analysis

Transcriptome data of tumor tissues from 10 HCC patients with different responses were obtained from our previous study [15]. For transcriptome data of neutrophils treated with CCL21, we extracted RNA from neutrophils using the Triazolo reagent (Invitrogen, USA) and sent to Agilent 2100 bioanalyzer for integrity check. After enrichment and random cleavage, fragmented messenger RNA (mRNA) was reversely transcribed into complementary DNA (cDNA) and further amplified by PCR to construct cDNA library. Subsequently, transcriptome sequencing was performed on a HiSeq X platform (Illumina). Besides, to explore the different pathways between treated and control groups, we performed gene set enrichment analysis (GSEA) in the GSEA software (version 4.3.2) using Hallmark and C2 (KEGG) gene sets downloaded from the MSigDB website. *P* value and FDR less than 0.05 were considered statistically significant.

### Single-cell RNA sequencing analyses

Single-cell RNA sequencing data (GSE125449 and GSE127645) were obtained from the Gene Expression Omnibus (GEO) website [16, 17]. Quality control steps included removal of doublets and cells with > 5% mitochondrial gene. Subsequently, after the transcriptome data were normalized and log-transformed, we detected 2000 highly variable genes for further principal component analysis based on the average expression and dispersion of the genes. UMAP v0.3.9 plots were used to visualize clusters of cells localized in the graph-based clusters by using ‘RunUMAP’ function. Markers for each cluster were identified by finding differentially expressed genes between cells from the individual cluster versus cells from all other clusters using the ‘FindAllMarkers’ function. Clusters were further annotated by ‘Enrichr’ software with the markers identified above [18].

### Quantitative real-time PCR

After extracted from cells as described above, RNA was reverse-transcribed into cDNA using the PrimeScript RT reagent kit (Takara, Japan) according to the standard procedure. A PCR reaction system was established using cDNA, primers, and SYBR Green Master Mix according to the manufacturer’s instructions and run on an ABI Prism 7500 Sequence Detection System (Applied Biosystems). The relative mRNA level of the target gene was calculated using the ∆∆Ct method with housekeeping gene GAPDH as control. The sequences of the primers are listed in Supplemental Table 1.

### Western blot assay

Proteins were extracted from cultured cells using radio-immunoprecipitation assay (RIPA) buffer and quantified using the Bicinchoninic Acid (BCA) assay kit. After thermal denaturation, proteins were separated by sodium dodecyl sulfate polyacrylamide gel electrophoresis (SDS-PAGE) and transferred onto the Polyvinylidene Fluoride (PVDF) membranes according to the standard procedure. Later, bands were blocked with 5% skim milk and incubated overnight with the corresponding primary antibody. Finally, the blots were exposed with chemiluminescence reagents after incubating with the HRP-conjugated secondary antibody. The primary antibodies are summarized in Supplemental Table 2.

### Flow cytometry

Cultured cells were trypsinized using trypsin and tumors harvested from mouse models were shredded and digested using collagenase to obtain single cell suspension. Later, after stained with fixable viability dye and permeabilized, cells were incubated with fluorochrome-conjugated antibodies in the dark for 30 min and further sent to the BD FACSAria III Flow Cytometer for flow cytometry. The results were analyzed using the FlowJo software. The antibodies used are shown in Supplemental Table 2.

### Animal studies

In vivo tumorigenesis assay was performed with male C57BL/6 J mice aged 6 weeks. Briefly, mouse HCC cells Hepa1-6 (1 × 10^6^) were subcutaneously injected into the right flank of each mouse. Once palpable, the following treatment commenced and mice were randomly divided into four groups (five mice per group): for CCL21 therapy, mice were injected intraperitoneally with two times per week doses of 0.25 μg recombinant murine CCL21 (Cat. 250-13, PeproTech) or vehicle control; for anti-PD-1 therapy, mice were treated intraperitoneally with two times per week doses of 100 µg anti-mouse PD-1 antibody (Cat. BP0273, Bio X Cell) or IgG isotype control. Tumor size was measured using vernier caliper and calculated as 0.5 × length × width^2^. All experiments were performed in accordance with the guidelines for care and use of laboratory animals and approved by the Zhongshan Hospital Institutional Animal Care and Ethics Committee, Fudan University.

### Statistical analysis

Numerical variables were shown as mean ± SD and differences between groups was tested using Student’s t- test, one-way analysis of variance or Mann–Whitney U test as appropriate. Categorical variables were presented as *n* (%) and constituent ratio between groups was compared using Pearson χ^2^ and Fisher’s exact test. All analyses were performed with SPSS V.25.0 (IBM, Armonk, New York, USA) and R software (V.4.2.2). A two-tailed *P* value less than 0.05 considered statistically significant.

## Results

### Association between CCL21 levels and immunotherapy response in HCC

**Fig. 1:**
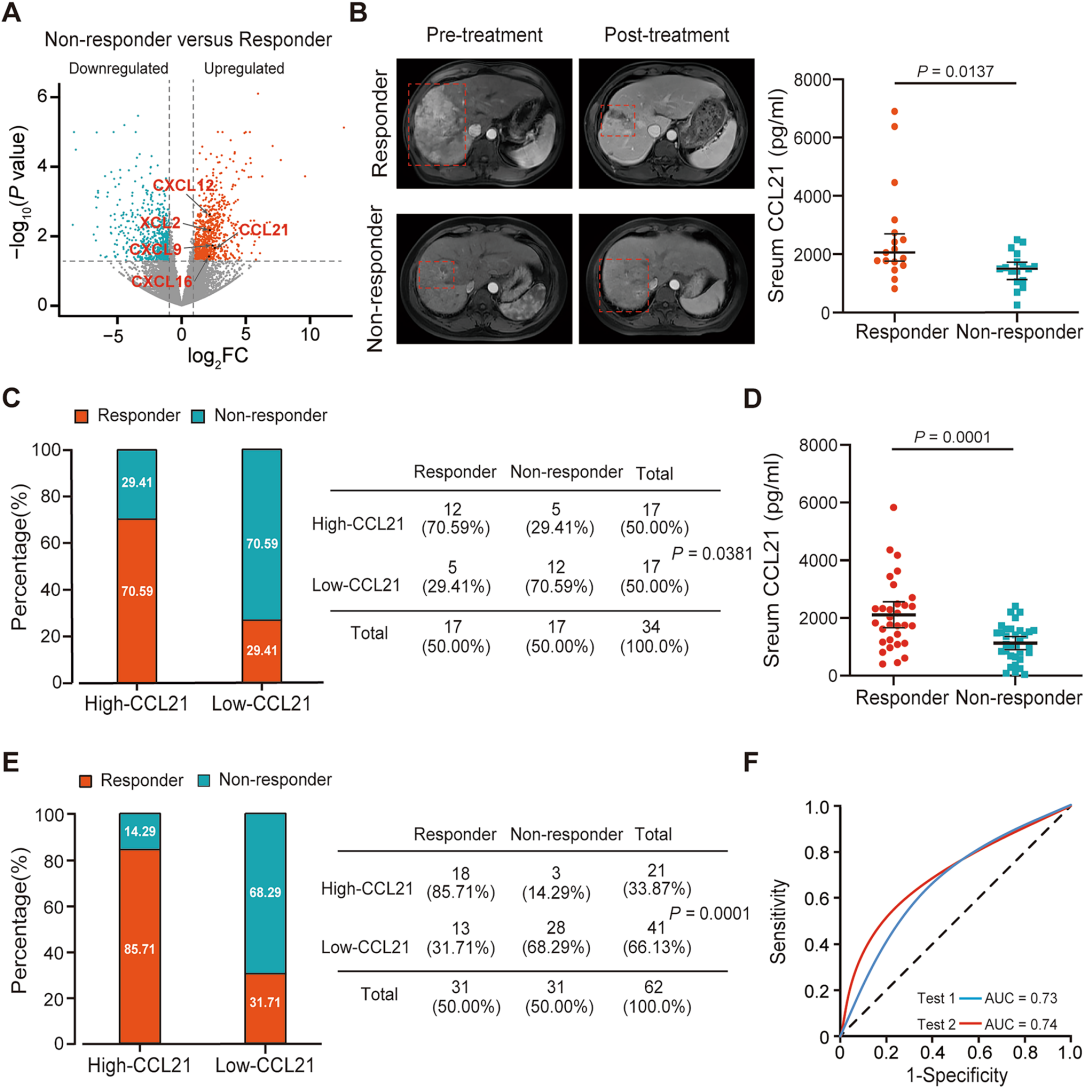
Association between CCL21 levels and immunotherapy response in HCC. **A** Transcriptome analysis showed that CCL21 was upregulated in responders. **B** Serum CCL21 levels of responders and non-responders in the training cohort. **C** Using the median serum CCL21 concentration (1736 pg/ml) as the cutoff value, the proportion of responders and non-responders in different serum CCL21 levels. **D** Serum CCL21 levels of responders and non-responders in the validation cohort. **E** Using the median serum CCL21 concentration of training cohort as the cutoff value, the proportion of responders and non-responders in different serum CCL21 levels. **F** ROC curve analysis of predictive performance of high serum CCL21 level for predicting immunotherapy response in training and validation cohorts. One-way ANOVA with a post hoc LSD test. Categorical variables were presented as *n* (%) and constituent ratio between groups was compared using Pearson *χ*^2^ and Fisher’s exact test. HCC, hepatocellular carcinoma; CCL21, Chemokine C–C motif ligand 21; ROC curve analysis, receiver operating characteristic curve analysis

To investigate potential differences in cytokine profiles among HCC patients with different responses to immunotherapy, we analyzed tumor tissues from 10 HCC patients with different responses for transcriptome analysis. We observed a statistically significant upregulation of CCL21 expression in the tumor tissues of HCC patients that responded to immunotherapy (Fig. 1A). To further explore the relationship between serum CCL21 levels and immunotherapy response in HCC patients, we employed a training cohort consisting of 34 HCC patients. We found that HCC patients that responded to immunotherapy had significantly higher levels of serum CCL21 compared to those with no response (Fig. 1B). Subsequently, the median concentration of serum CCL21 (1736 pg/ml) was utilized as the cutoff value to divide the training group into high and low CCL21 groups. We observed that the high CCL21 group had a greater proportion of patients with response to immunotherapy compared to the low CCL21 group (Fig. 1C). Similar results were observed in the validation cohort containing 62 patients when using the same cutoff values (Fig. 1D, E). In addition, we performed ROC curve analysis to determine the predictive performance of serum CCL21 levels in predicting immunotherapy response. The AUC values for the training and validation cohorts were 0.73 (95% CI 0.56–0.90) and 0.74 (95% CI: 0.64–0.84), respectively (Fig. 1F). C–C chemokine receptor 7 (CCR7) was considered to be the principal receptor for CCL21 [19–21]. However, we found that there was no significant difference in CCR7 among HCC patients with different responses to immunotherapy by transcriptomic and experimental analyses (Supplemental Fig. 1A, B). Besides, CCL21 expression was independent on tumor stage (Supplemental Fig. 1C). Through employing a public data set (GSE125449) containing single-cell RNA sequencing data of 19 patients with liver cancer for bioinformatics analyses, we found that CCL21 in liver cancer was mainly derived from stromal cells like fibroblasts and epithelial cells (Supplemental Fig. 1D–F). Overall, these results suggest that CCL21 can serve as a potential predictive biomarker for immunotherapy response in HCC.

### Construction of a predictive nomogram for tumor immunotherapy response

**Fig. 2:**
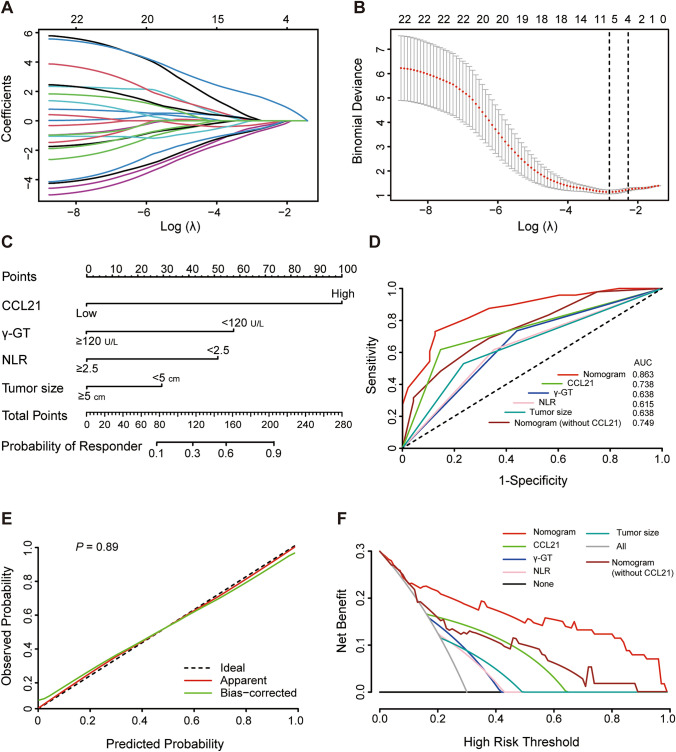
Construction of a predictive nomogram for tumor immunotherapy response. **A** and **B** LASSO analysis and 1000-fold cross-validation were used to identify the most valuable predictors associated with response to immunotherapy in HCC. **C** Nomogram for pre-treatment prediction of immunotherapy response. **D** ROC curve analysis of predictive performance of independent predictors and nomogram for predicting immunotherapy. **E** Calibration curves for the consistency between actual observation and nomogram prediction. **F** DCA for the net benefit of the nomogram and independent predictors. LASSO, least absolute shrinkage and selection operator; HCC, hepatocellular carcinoma; DCA, decision curve analysis

Considering the close relationship between CCL21 levels and immunotherapy response in HCC, we further attempted to investigate whether CCL21 could be integrated into existing markers to optimize immunotherapeutic management of HCC. We combined the above two HCC patient cohorts and collected a total of 22 clinicopathological variables (Supplemental Table 3), from which we screened the four most useful predictors including tumor size, gamma-glutamyl transferase (γ-GT), neutrophil to lymphocyte ratio (NLR) and CCL21, with non-zero coefficients in the LASSO regression model (Fig. 2A, B and Supplemental Table 4). These candidate variables were then incorporated to construct a predictive nomogram for tumor immunotherapy response (Fig. 2C). The predictive nomogram showed a better discrimination compared to these independent predictors alone with an AUC value of 0.863 (95% CI 0.790–0.935) (Fig. 2D). Meanwhile, calibration curves demonstrated good consistency between actual observation and nomogram prediction (Fig. 2E). The Hosmer–Lemeshow test (*p* = 0.89) also indicated that the nomogram had a superior fit. The optimal cutoff value for the Nomo-score was 0.25. The sensitivity and specificity of nomogram were 87.5% and 72.9%, respectively. Additionally, DCA with a threshold probability of 30% was used to calculate the net benefit of the nomogram, and results showed that the nomogram had higher net benefit than independent predictors (Fig. 2F). Therefore, the CCL21-based predictive nomogram we constructed is clinically important for selecting appropriate treatment strategies for HCC patients.

### CCL21 inhibits neutrophil N2 polarization through regulating NF-κB signaling pathway

Given the potential role of CCL21 as a cytokine in the remodeling of TME, we speculate that the association of CCL21 with immunotherapeutic response in HCC is likely to be related to its impact on the immune microenvironment. To test this hypothesis, we first performed ssGSEA based on TCGA-LIHC cohort to determine the relative abundance of immune cells in tumor tissues with different CCL21 expression level. The results showed that tumors with high CCL21 expression tended to have more infiltrations of immune cells, suggesting higher immunoreactivity compared to those with low CCL21 level (Supplemental Figs. 2A, B). In addition, CIBERSORT algorithm showed that CCL21 levels were strongly and positively correlated with various immune cell types including neutrophils, CD8^+^ T cells, and macrophages (Supplemental Fig. 2C). Collectively, the findings show that low CCL21 levels may induce an immune exhaustion contexture in HCC.

**Fig. 3:**
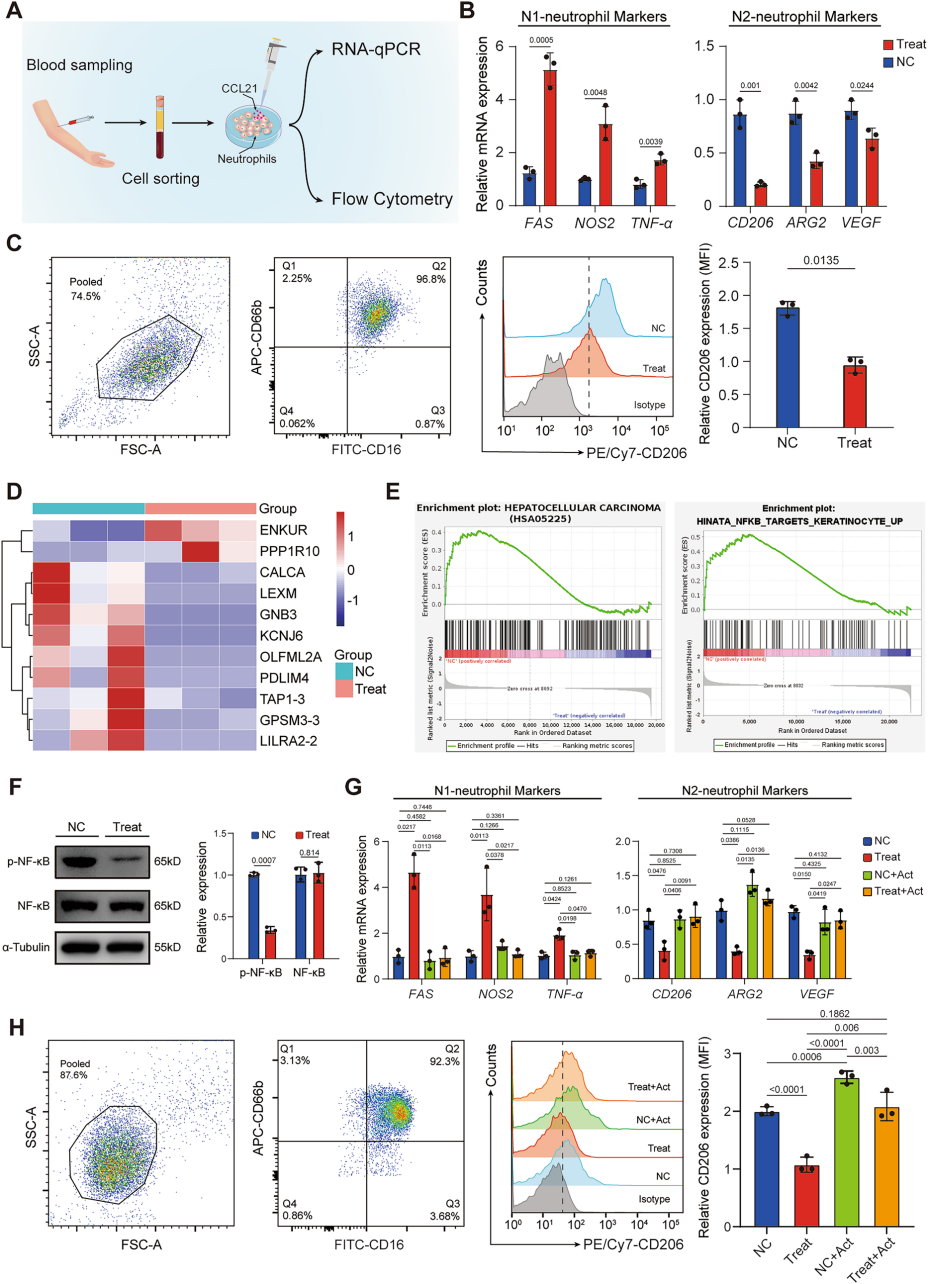
CCL21 inhibits neutrophil N2 polarization through regulating NF-κB signaling pathway. **A** Schematic diagram of neutrophil isolation and culture in vitro. **B** qPCR analysis of the transcription of N1 and N2 markers in neutrophils treated with or without recombinant human CCL21. **C** Flow cytometry analysis of CD206 expression of neutrophils in indicated groups. **D** Heatmap of the differential genes in negative control and CCL21 treatment groups. **E** Differentially enriched pathways in negative control and CCL21 treatment groups identified by GSEA. **F** Western blot of NF-κB pathway in neutrophils treated with or without recombinant human CCL21. **G** qPCR analysis of the transcription of N1 and N2 markers in neutrophils treated with or without recombinant human CCL21 and/or NF-κB activator 1. **H** Flow cytometry analysis of CD206 expression of neutrophils in indicated groups. One-way ANOVA with a post hoc LSD test. CCL21, Chemokine C–C motif ligand 21; qPCR, quantitative real- time PCR; GSEA, gene set enrichment analysis; NF-κB, nuclear factor kappa B

Extensive research has been conducted to elucidate the role of CCL21 in T cells and macrophages [22, 23], yet its effects on neutrophils remain unclear. Therefore, we next focused on the influence of CCL21 on neutrophil functional polarization. We collected blood samples from healthy individuals to isolate neutrophils and then treated these neutrophils with recombinant human CCL21. Subsequently, RNA-qPCR and flow cytometry were performed for experimental analysis (Fig. 3A). RNA-qPCR results showed that CCL21 increased the expression of N1 neutrophil markers (such as FAS, NOS2, TNF-α) and decreased the expression of N2 neutrophil markers (such as CD206, ARG2, VEGF), which was further validated in the flow cytometry (Fig. 3B, C). We next performed transcriptome analysis and GSEA, and found that ‘HEPATOCELLULAR CARCINOMA’ and ‘HINATA_NFKB_TARGETS_KERATINOCYTE_UP’ gene signatures were significantly enriched in control group (Fig. 3D, E). A study has revealed the involvement of nuclear factor kappa B (NF-κB) pathway in the N2 neutrophils polarization [24]. Through Western blot assay, we verified that CCL21 inhibited the activation of the NF-κB pathway. To investigate whether CCL21 inhibits N2 neutrophil polarization through regulating the NF-κB pathway, we treated neutrophils with recombinant human CCL21 and/or NF-κB activator 1. The results showed that NF-κB activator 1 reversed the effect of CCL21 on neutrophil N1 polarization, which was further verified in flow cytometry (Fig. 3G, H). Given that CCR7 is the principal receptor for CCL21, we performed bioinformatic analysis by employing a public data set (GSE127645) containing single-cell RNA sequencing data. The expression of CCR7 was not associated with neutrophil phenotype (Supplemental Fig. 2D). Overall, these findings suggested that CCL21 inhibits N2 neutrophil polarization through regulating NF-κB pathway.

### CCL21 enhances the efficacy of anti-PD-1 antibody in HCC in vivo

**Fig. 4:**
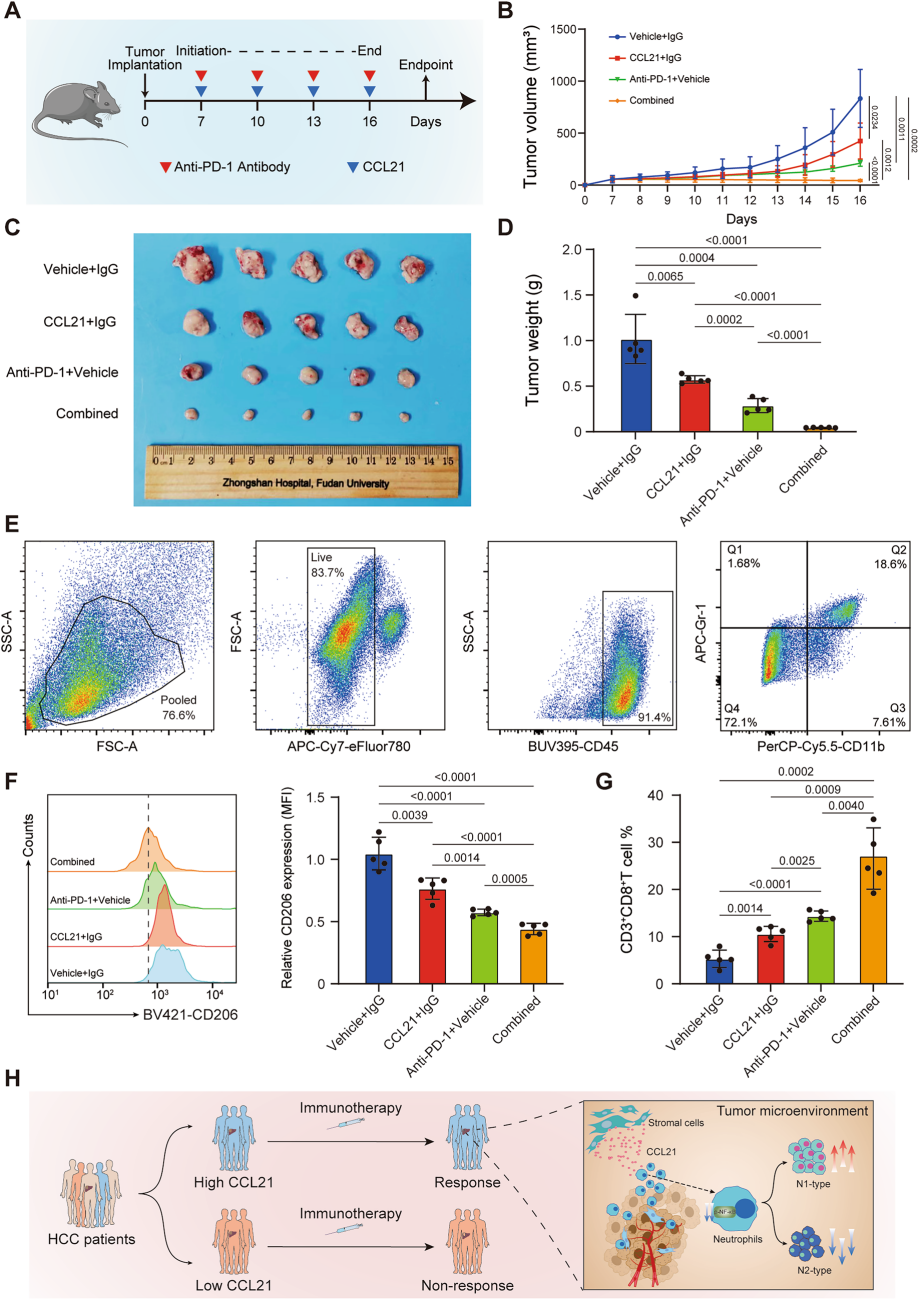
CCL21 enhances the efficacy of anti-PD-1 antibody in HCC in vivo. **A** Schematic diagram of anti-PD-1 antibody and recombinant murine CCL21 treatment in the mice model of HCC subcutaneous tumors. **B** The volume of subcutaneous tumors in each group during treatment. **C** Gross appearance of the subcutaneous HCC tumors from the indicated treatment groups. **D** The weight of subcutaneous tumors in each group at the endpoint. **E**–**G** Flow cytometry analyses of CD206 expression of neutrophils and the tumor infiltration percentage of CD3^+^CD8^+^ T cells in each group. **H** Schematic diagram depicting that CCL21 determines immunotherapy response in HCC by affecting neutrophil polarization. One-way ANOVA with a post hoc LSD test. CCL21, Chemokine C–C motif ligand 21; PD-1, programmed death receptor 1; HCC, hepatocellular carcinoma

The above results drove us to investigate whether CCL21 could enhance the therapeutic efficacy of anti-PD-1 antibody in HCC. We constructed Hepa1-6-derived subcutaneous HCC mouse models and treated them with IgG, anti-PD-1 antibody, recombinant murine CCL21, and anti-PD-1 antibody combined with CCL21, respectively (Fig. 4A). During treatment, CCL21, anti-PD-1 antibody and combination therapy all significantly restricted the growth of subcutaneous tumors (Fig. 4B). In addition, tumors harvested at the endpoint of experiment were much smaller for combination therapy than monotherapies (Fig. 4C, D). To further understand the mechanism underlying the enhanced anti-tumor efficacy of combined therapy, we dissected the tumor immune infiltration landscape of Hepa1-6 subcutaneous tumors. As shown by flow cytometric analyses, mice administered with combination therapy had significantly decreased tumor infiltrations of N2 neutrophils and increased tumor infiltrations of CD8^+^ T cells compared with the monotherapies and the control (Fig. 4E–G). Collectively, these findings stressed that CCL21 may enhance the therapeutic efficacy of anti-PD-1 therapy in HCC.

## Discussion

Immunotherapy, represented by ICIs, has brought a breakthrough in the clinical treatment of HCC. However, the lack of biomarkers that can predict patient response or improve the efficacy of immunotherapy remains the biggest obstacle. We analyzed the transcriptome data of tumor tissues from 10 HCC patients with different responses and the results showed that CCL21 expression was upregulated in patients with response to immunotherapy. Furthermore, ELISA performed in our own patient cohorts highlighted that serum CCL21 levels had great predictive capabilities in immunotherapy response. Subsequently, we integrated six independent predictors to construct a CC21-based predictive nomogram. ROC curve analysis and DCA showed better discrimination and more net benefit of the nomogram compared to those independent predictors. As for other predictive models in HCC, Luo, et al. [25] constructed a 5-gene (NET1, ATP6V0B, MMP1, GTDC1, and CPEB3) risk model through LASSO analyses for predicting immunotherapy response. Feng, et al. [26] identified 17 immune-related gene pairs to explore its predictiveness to ICIs via the LASSO algorithm. In comparison with the other models, our predictive nomogram shows stronger clinical applicability and predictive values. In addition, previous studies have showed that tumor tissue PD-L1 expression levels [27] and tumor mutation burden [28, 29] are potentially biomarkers for immunotherapy response prediction. However, due to the unavailability of advanced HCC tissues, these biomarkers presented significant challenges in assessing immunotherapy response in HCC patients. In this study, we highlight that serum CCL21 offers a non-invasive approach, providing patients with a more comfortable and safer option. Despite the potential risk of overfitting caused by the small sample in our model, the predictive performance of the model is excellent from our results. In summary, serum CCL21 can serve as a potential predictive biomarker to enable physicians to forecast patients’ response to immunotherapy, which may facilitate individualized treatment strategies and allow dynamic monitoring of patients.

CCL21, as an important chemokine, has diverse effects in the regulation of immune cells. Numerous studies have shown that CCL21 plays an important role in T cell infiltration and macrophage polarization [23, 30]. Our study further revealed that CCL21 not only had effects on T cells and macrophages, but also exerted significant regulatory effects on neutrophil polarization. The results showed that CCL21 could inhibit the polarization of N2 neutrophils while promoting the polarization of N1 neutrophils. In addition, a study suggested that NF-κB pathway had a crucial regulatory role in N2 neutrophil polarization [24]. In our study, we validated this finding and further demonstrated that CCL21 inhibits N2 neutrophil polarization by affecting the activation of NF-κB pathway. These results provide new insights into the mechanism for CCL21 regulation of neutrophil function.

The above results demonstrated that CCL21 could inhibit immunosuppressive neutrophil polarization in the TME of HCC, and high levels of CCL21 in the TME were characterized by high infiltration of macrophages and CD8^+^ T cells. Given the features of tumor immune infiltration have been acknowledged as pivotal factors impacting the response to immunotherapy [31], we hypothesized that CCL21 might potentiate the efficacy of ICIs in HCC. The enhanced anti-tumor effect of the combination of anti-PD-1 antibody and CCL21 in our vivo experiments fully supports this hypothesis. A study in pancreatic cancer reported that CCL21 could enhance T cell-mediated cytotoxicity and the efficacy of ICIs [32]. Therefore, based on our findings, adjuvant CCL21 therapy disrupt the role of neutrophils in forming an immunosuppressive TME and enhance the therapeutic efficacy of immunotherapy in HCC, which has great clinical translational value.

However, given the retrospective study design, potential biases and the relatively small sample size, it is required to conduct prospective studies with large-scale, multicenter patient cohorts to validate our findings. Besides, considering the present study did not elucidate specific regulatory mechanisms for CCL21 affecting NF-κB pathway to regulate neutrophils as well as the potential effects of CCL21 on tumor cells, basic studies based on in vivo and in vitro experiments are needed in the future. The therapeutic efficacy of CCL21 combined with ICIs in HCC also requires further investigation.

## Conclusion

In conclusion, our study suggests that CCL21 may serve as a potential predictive biomarker for immunotherapy response in HCC patients. High levels of CCL21 inhibits N2 neutrophils polarization and promotes N1 neutrophils polarization, which transforms immunosuppressive TME into an anti-tumor context (Fig. 4H). These findings provide a novel HCC therapeutic strategy of CCL21 in combination with immunotherapy.

### Supplementary Information

Below is the link to the electronic supplementary material.

Supplementary file1 (DOCX 1839 KB)

## Data Availability

All data relevant to the study are included in the article or uploaded as supplementary information.
