# Supplementary material for: Chemokine CCL21 determines immunotherapy response in hepatocellular carcinoma by affecting neutrophil polarization
Source: Cancer Immunol Immunother. 2024 Feb 17;73(3):56. doi: 10.1007/s00262-024-03650-4 (PMC10874310; doi:10.1007/s00262-024-03650-4)
Supplement: Supplementary file 1 — Supplementary file1 (DOCX 1839 KB) [file 262_2024_3650_MOESM1_ESM.docx]

**Supplemental Figures**

**
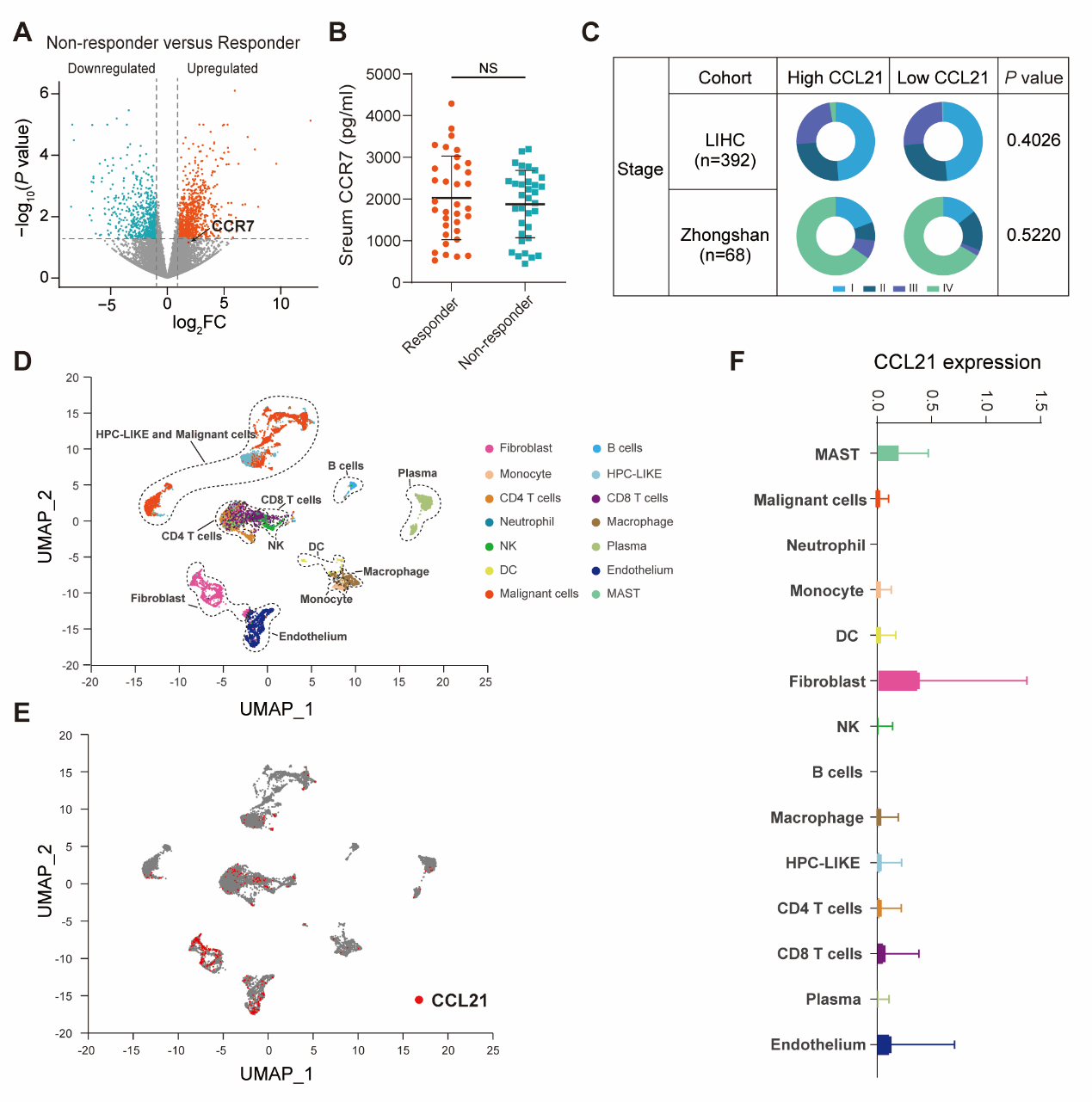
**

**Supplemental Fig. 1 CCR7 was not associated with immunotherapy, and CCL21 in liver cancer was mainly derived from stromal cells.** (A) Transcriptome analysis showed that CCR7 was no significant in responders. (B) Serum CCR7 levels of responders and non-responders in our patients cohort. (C) The circular pie chart shows the proportion difference of tumor stage in the high CCL21 and low CCL21 expression groups from the TCGA-LIHC cohort and our patients cohort. (D) UMAP plot of 9716 single cells from 19 patients with liver cancer in GSE125449 cohort. (E) UMAP plot shows the cells expressed CCL21. (F) Histogram shows the expression level of CCL21 in different cell types. CCL21, Chemokine C‐C motif ligand 21; TCGA-LIHC, cancer genome atlas liver hepatocellular carcinoma.

**
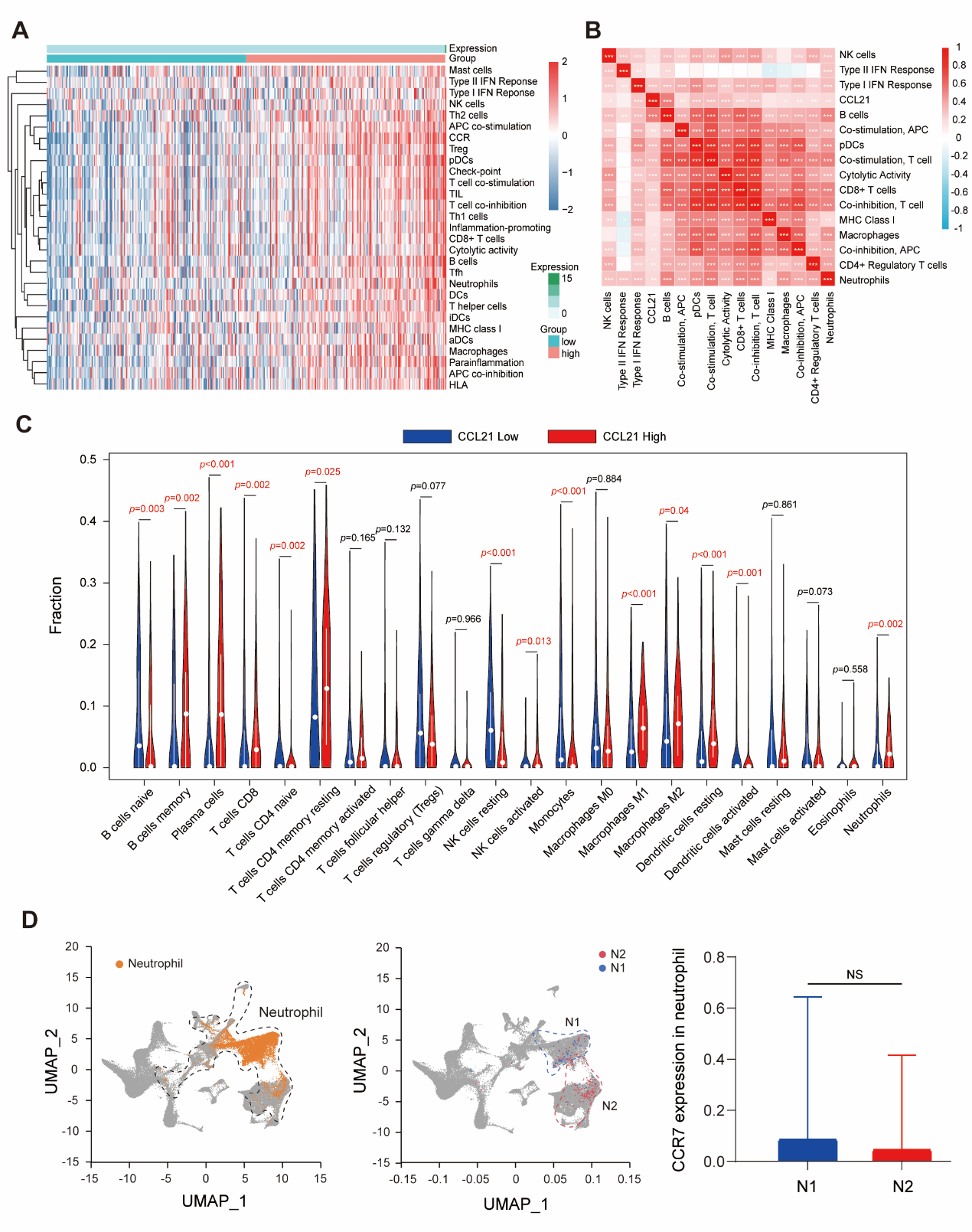
**

**Supplemental Fig. 2 High CCL21 expression was associated with high immune infiltration in TME of HCC.** (A) Heatmap of immunological activity score calculated by ssGSEA in each tumor sample in TCGA-LIHC cohort based on the clustering analysis. (B) Correlation matrix of the score and immune cell types. (C) Violin chart of the infiltration percentage of 22 immune cell types in the CCL21 high and low expression groups calculated by CIBERSORT algorithm. (D) UMAP plot shows the expression of CCR7 in N1 and N2 neutrophil. CCL21, Chemokine C‐C motif ligand 21; HCC, hepatocellular carcinoma; TME, tumor microenvironment; ssGSEA, single-sample gene set enrichment analysis; TCGA-LIHC, cancer genome atlas liver hepatocellular carcinoma; CIBERSORT, cell-type identification by estimating relative subsets of RNA transcripts.

**Supplemental Tables**

**Supplemental Table 1. Sequences of primers (5’-3’) used for qRT-PCR.**

| Genes | | Sequences (5’-3’) |
| --- | --- | --- |
| Human FAS | FORWARD | GACCCTCCTACCTCTGGTTCTT |
| Human FAS | REVERSE | CTGGAGGACAGGGCTTATGG |
| Human NOS2 | FORWARD | CGTGGAGACGGGAAAGAAGT |
| Human NOS2 | REVERSE | GACCCCAGGCAAGATTTGGA |
| Human TNF-α | FORWARD | TCTCCTTCCTGATCGTGGCA |
| Human TNF-α | REVERSE | CAGCTTGAGGGTTTGCTACAAC |
| Human CD206 | FORWARD | ATTCAGATATGCCAGGGCGA |
| Human CD206 | REVERSE | ATTTGGGTTCGGGAGTCGTC |
| Human ARG2 | FORWARD | TTCTCAGTGCTGCGGATCATGT |
| Human ARG2 | REVERSE | GCTCCACTCCTTTTCTTTTCTGCC |
| Human VEGF | FORWARD | CAACAAATGTGAATGCAGACCAA |
| Human VEGF | REVERSE | GCTCCAGGGCATTAGACAGC |
| Human β-actin | FORWARD | ACCAACTGGGACGACATGGAGAAA |
| Human β-actin | REVERSE | TAGCACAGCCTGGATAGCAACGTA |

**Supplemental Table 2. Primary antibodies used in the study**

| Antibody | Concentration | Application | Identifier | Company |
| --- | --- | --- | --- | --- |
| Anti-human NF-κB | 1:1000 | Western blot | Cat#8242 | Cell Signaling Technology |
| Anti-human Phospho-NF-κB | 1:1000 | Western blot | Cat#3033 | Cell Signaling Technology |
| α-Tubulin (11H10) | 1:1000 | Western blot | Cat#2125 | Cell Signaling Technology |
| FITC anti-human CD16 | 5µl/million cells | Flow cytometry | Cat#302005 | Biolegend |
| APC anti-human CD66b | 5µl/million cells | Flow cytometry | Cat#305117 | Biolegend |
| PE/Cyanine7 anti-human CD206 (MMR) | 5µl/million cells | Flow cytometry | Cat#321124 | Biolegend |
| Brilliant Violet 421™ anti-mouse CD206 (MMR) | 5µl/million cells | Flow cytometry | Cat#141717 | Biolegend |
| PerCP/Cyanine5.5 anti-mouse/human CD11b | 5µl/million cells | Flow cytometry | Cat#101227 | Biolegend |
| APC anti-mouse Ly-6G/Ly6C (Gr-1) | 5µl/million cells | Flow cytometry | Cat#108411 | Biolegend |
| PerCP/Cyanine5.5 anti-mouse CD8a | 5µl/million cells | Flow cytometry | Cat#100733 | Biolegend |
| PE/Cyanine5.5 anti-mouse CD3Ɛ | 5µl/million cells | Flow cytometry | Cat#100320 | Biolegend |
| eBioscience™ Fixable Viability Dye eFluor™ 780 | 5µl/million cells | Flow cytometry | Cat#2633407 | invitrogen |
| BUV395 Rat Anti-Mouse CD45 | 5µl/million cells | Flow cytometry | Cat#565967 | BD Bioscience |

**Supplemental Table 3.** **Characteristics of all HCC patients.**

| **Characteristic** | **HCC Patients (n)** |
| --- | --- |
| Age (≥60/<60, year) | 84/12 |
| Gender (Male/Female) | 85/11 |
| CCL21 (High/Low) | 38/58 |
| MVI (Yes/No) | 28/49 |
| Metastasis (Yes/No) | 14/82 |
| Cirrhosis (Yes/No) | 34/62 |
| Tumor number (Single/Multiple) | 57/39 |
| Tumor size (≥5/<5, cm) | 61/35 |
| γ-GT (≥120/ <120, U/L) | 41/55 |
| NLR (≥2.5/<2.5) | 48/48 |
| PIVKA-II (≥40/<40, mAU/ml) | 79/17 |
| PLR (≥100/<100) | 36/60 |
| AFP (≥20/<20, ng/ml) | 62/34 |
| CA199 (>40/≤40, U/ml) | 16/80 |
| AST (≥40/ <40, U/L) | 44/52 |
| ALT (≥40/ <40, U/L) | 39/57 |
| ALB (≥40/ <40, g/L) | 80/16 |
| HBsAg (Positive/Negative) | 73/23 |
| TB (≥20/ <20, μmol/L) | 44/52 |
| DB (≥7/ <7, μmol/L) | 38/58 |
| BCLC stage (A/B+C) | 17/79 |
| Child-Pugh class (A/B) | 9/87 |
| Tumor response, according to RECIST v1.1 (CR/PR/SD/PD) | 16/32/16/32 |

**Supplemental Table 4. LASSO regression analysis**

| Variable | β | OR (95%CI) | *P* Value |
| --- | --- | --- | --- |
| CCL21 | 1.18 | 21.85(5.32-151.42) | <0.001 |
| High vs. Low |  |  |  |
| γ-GT, U/L | -0.42 | 0.11(0.01-0.69) | 0.01 |
| ≥120 vs. <120 |  |  |  |
| NLR | -0.24 | 0.19(0.03-0.82) | 0.02 |
| ≥2.5 vs. <2.5 |  |  |  |
| Tumor size, cm | -0.28 | 0.21(0.01-1.07) | 0.02 |
| ≥5 vs. <5 |  |  |  |
